# The Effectiveness of a Healthy Lifestyle in Obese Pediatric Patients: A Systematic Review and Meta-Analysis

**DOI:** 10.7759/cureus.48525

**Published:** 2023-11-08

**Authors:** Andrea M Zuñiga Vinueza, Arturo P Jaramillo

**Affiliations:** 1 School of Medicine, Universidad Catolica de Santiago de Guayaquil, Guayaquil, ECU; 2 General Practice, Universidad Estatal de Guayaquil, Machala, ECU

**Keywords:** physical activities, obese children, diet modification, lifestyle diseases prevention, community obesity

## Abstract

Child and adolescent obesity represents a significant and escalating health concern in the United States. Notably, Hispanic adolescents face a higher prevalence of obesity and an increased risk of cardiovascular disease compared to their peers from different racial and ethnic backgrounds. This was obtained through systematic investigations in which different approaches were used. Therefore, obesity interventions of long duration, at least one year, and with a beginning phase intensive enough to produce significant early weight loss may be needed for adolescents with obesity. Surprisingly, despite this elevated risk, there is a glaring underrepresentation of Hispanics in obesity intervention studies aimed at youth. It is therefore imperative to develop interventions tailored specifically to overweight adolescents, with a particular focus on the Hispanic population. While researchers have addressed numerous interventions targeting adolescent obesity, many of these initiatives have demonstrated limited treatment efficacy, failed to achieve all desired treatment objectives, experienced high attrition rates, and encountered waning participant engagement. To evaluate the impact of adopting a healthy lifestyle among pediatric patients struggling with obesity, we undertook a comprehensive systematic review of the literature, and with the information obtained from the articles chosen, we will undergo a meta-analysis. Our review encompassed a 10-year span of published literature, drawing upon online databases including the Cochrane Library, PubMed, Web of Science, PubMed Central, and Google Scholar.

Our review exclusively considered randomized controlled trials that focused on the effectiveness of various lifestyle modifications for pediatric patients grappling with obesity. We synthesized the pooled incidence, risk ratio, and associated 95% confidence intervals to gauge the efficacy of these interventions, employing the fixed-effect model to account for potential between-study variations rather than the random-effect model. After the calculation of each one of the studies selected, we could conclude that it gave good outcomes after the modification of lifestyle in these patients, giving a statistical significance and p-value in our three representative figures of <0.001.

## Introduction and background

Childhood obesity has emerged as a growing concern, and recent data indicate that this issue is increasingly affecting young children. According to national data in 2017-2018, 13% of children aged 2-5 years in the United States are now grappling with overweight and obesity, representing a significant increase from 8% in 2011-2012 [1]. Several factors contribute to these disparities in childhood obesity rates, including lower household income, rural residence, and low socioeconomic status at the community level [2-5]. The preschool years are a critical stage for implementing preventive measures because substantial increases in body mass index (BMI) during this period can lead to overweight, obesity, and various associated health complications [6-8]. These health issues encompass adverse metabolic and cardiovascular outcomes, such as high dyslipidemia, elevated blood pressure, and insulin resistance [8]. Moreover, obese children may experience other adverse physical health effects, including a reduced quality of life and lower self-esteem regarding their health when compared to their peers with normal weight [9,10].

Childhood obesity has enduring consequences. Longitudinal studies have shown that a significant proportion, ranging from 60% to 90%, of children diagnosed with obesity during their preschool years continue to grapple with this condition into adolescence and adulthood [11,12]. The persistence of obesity into adulthood can profoundly impact an individual’s health and significantly affect the overall well-being of the population. In the United States in 2016, data on the high prevalence of obesity in children between 2 and 19 years suggest that approximately 57% of the population is likely to develop obesity before reaching the age of 35 [13]. This could result in a significant portion of the population facing health issues related to obesity [13,14].

To tackle the multifaceted public health challenge of childhood obesity, primary prevention approaches are essential. Primary care clinics provide a sustainable platform for delivering interventions through well-child visits (WCVs), especially because parents of young children highly value and rely on the guidance provided by pediatricians [15]. While primary care providers (PCPs) play a central role in obesity prevention, the effectiveness of clinical preventive care in addressing childhood obesity has been limited. Despite the majority of children participating in WCVs, the prevalence of obesity in preschool-age children remains a significant concern [1].

PCPs have the potential to promote healthy habits and connect families with community resources, but the lack of research on effective strategies for preventing childhood obesity in preschool-age children has impeded progress in this area [16]. The use of patient-reported outcome (PRO) measures in clinical care offers an opportunity to enhance patient-centered care by involving parents in discussions about preventive measures. Patients can achieve this by recording their own results. The widespread use of PRO measures allows for the collection of data directly from patients about their experiences, perceptions, or beliefs related to specific health conditions or outcomes [17]. Recent advancements in health information technology have made it possible to offer PRO measures in user-friendly formats accessible through online patient portals or clinic-provided tablets. These responses can be seamlessly integrated into patients’ electronic health records, streamlining the assessment by healthcare providers and enhancing patient-centered care [17].

One effective way to improve the efficacy of WCVs is by incorporating PRO measures, a practice that enables parents to assess their behaviors, practices, and home environments that may contribute to obesity. By identifying these factors early on, it becomes possible to predict the risk of obesity before it becomes a problem [17]. In a previous study, the use of a reliable questionnaire, the Family Nutrition and Physical Activity Questionnaire, demonstrated effectiveness in preventing obesity in preschool-age children. This study, conducted within a large health system, involved systematic data collection during WCVs [17]. Prior research has shown that involving parents in family-centered health coaching related to nutrition and physical activity, along with connecting them with community resources such as grocery store tours after WCVs, can effectively prevent childhood obesity. This approach also promotes food resource management to address food insecurity in low-income families [17].

In summary, the available evidence suggests that the integration of PRO measures, health coaching, and community referrals to WCVs holds promise for preventing childhood obesity. However, it is important to note that conclusive evidence is still needed regarding the most effective models for implementing these interventions in real-world settings [17].

## Review

Methodology

Study Duration and Search Strategy

On August 15, 2023, we started our systematic review by taking five databases, which were PubMed Central, PubMed, Cochrane Library, and Web of Science. After the selection of the corresponding articles, we established them based on the PRISMA flow diagram guidelines [18]. With this, we assure that everything will be clear and understandable. During our search in each database using Boolean logic "NOT", "AND", and "OR" to enhance the specificity and sensitivity of our search and MeSH keywords, we were reassuring the most reliable articles related to our title. These keywords and search strategies are elucidated in Table 1.

**Table 1:** Detailed literature search strategy

Search Strategy	Databases Used	Number of Papers Identified
Obesity AND Sendentarism AND Unhealthy habits	The Cochrane Library	27
( "Pediatric Obesity/complications"[Majr] OR "Pediatric Obesity/congenital"[Majr] OR "Pediatric Obesity/diet therapy"[Majr] OR "Pediatric Obesity/drug therapy"[Majr] OR "Pediatric Obesity/epidemiology"[Majr] OR "Pediatric Obesity/genetics"[Majr] OR "Pediatric Obesity/prevention and control"[Majr] OR "Pediatric Obesity/rehabilitation"[Majr] OR "Pediatric Obesity/therapy"[Majr] )	PubMed	227
"Obesity[tw]" AND "Sedentarism[tiab]" AND "Sendentarism[all]"	Google Scholar	82
Obesity or Sedentarism or Unhealthy habits, Obesity and Sedentarism and Unhealthy habits	PubMed Central	30
Obesity or Sedentarism or Unhealthy habits, Obesity and Sedentarism and Unhealthy habits	Web of Science	57

Study Selection

Once we chose the free-full-length articles, we underwent the Cochrane quality assessment tool, thus avoiding any potential bias from the 10 chosen articles and giving our research article better credibility and robustness. Randomized controlled trials (RCTs) that satisfied the effectiveness of the lifestyle modification in obese pediatric patients were considered. Two researchers thoroughly studied the titles and abstracts of all relevant RCTs to determine their eligibility. We focused on recent works from the last decade, prioritizing papers originally authored in English or with a freely accessible English translation. If a full version of the manuscripts containing the RCTs could not be found, they were disqualified. Only RCTs that specifically examined the results of implementing a lifestyle change for pediatric patients with obesity were included. Proposal papers and gray literature were also left out.

Statistical Analysis

2020: The Cochrane Collaboration, The Nordic Cochrane Centre, Copenhagen, Denmark RevMan version 5.4 was utilized for all statistical analyses. Other features were added like Mantel-Haenszel to calculate the standard errors or standard deviations, odds ratio (OR) effect calculation, a confidence interval (CI) of 95%, and we use a fixed-effect (FE) model due to the possibility of having a high study variance due to the population and study designs rather than a random-effect (RE) model.

The combined data was visually analyzed using forest plots. The chi-square test was used to identify statistically significant differences between the categories. The degree of heterogeneity (I^2^) in the studies was quantified using the Higgins I^2^, with values below 50% being deemed appropriate. The funnel plot was used for a visual assessment of publication bias. Each instance was taken into consideration at a significance level lower than 0.05.

Results

Search Results

**Figure 1:**
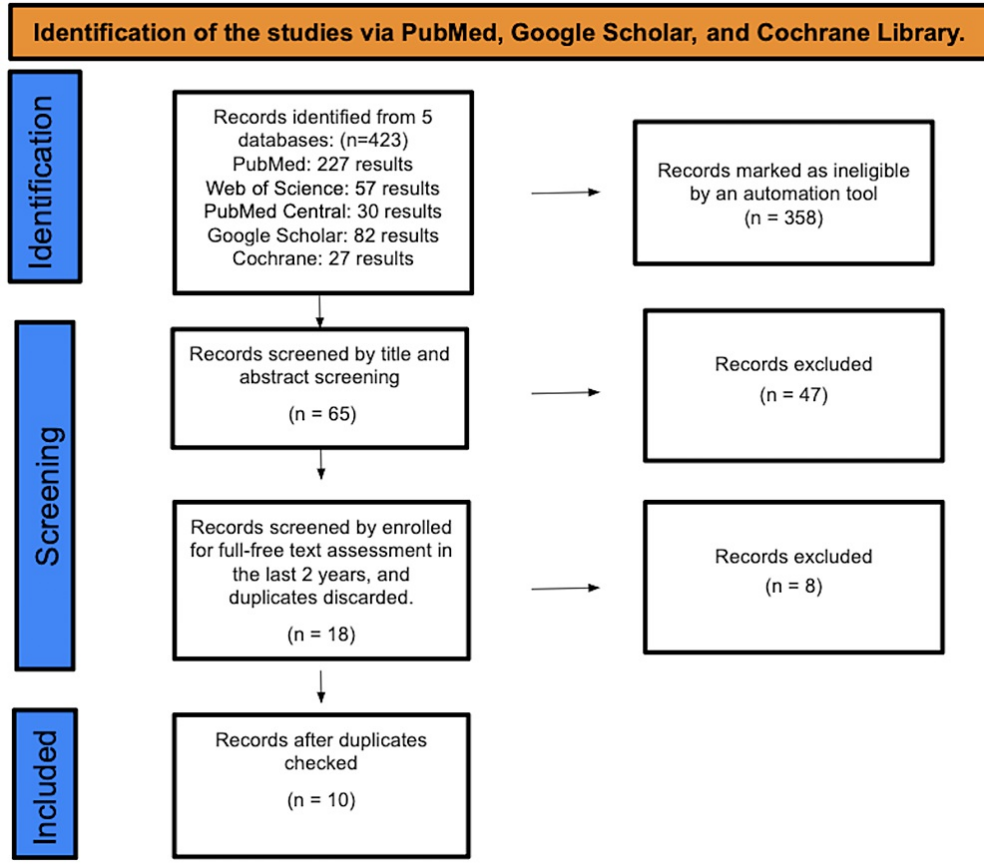
PRISMA flow diagram

After looking through results from PubMed Central, PubMed, Cochrane Library, and Web of Science, we came up with a total of 423 papers. An automated system rejected 358 of them as unreliable. The screening process began with the abstracts and titles of 65 study findings and resulted in the elimination of 47 articles. After eliminating duplicates (which led to the removal of eight research studies), the remaining 18 publications were selected using full-text assessment from the preceding decade, leaving just 10 studies for the final data collection. Database and registry searches for research are illustrated in Figure 1.

See Table 2 for an overview of the selected articles.

**Table 2:** Table of data extraction RCT: randomized controlled trial; BMIZ: body mass index z‑score; WHO: World Health Organization; WCV: well-child visit; FCUHealth: Family Check-Up 4 Health; BMI-SDS: BMI standard deviation score; BMI: body mass index; SWITCH: Screen Time Weight-loss Intervention Targeting Children at Home; CDS: clinical decision support; SDC: soft drinks; PA: physical activity.

Author	Year of Publication	Study Design	Quality Tool	Primary Research	Outcome Evaluation
Bailey et al. [17]	2022	RCT	Cochrane risk of bias assessment tool	Using 24 primary care clinics at Geisinger Health System, we enrolled 2,025 parents and their children between the ages of 20 and 60 months.	Twelve months after baseline WCV, WHO growth standards-based child BMI change is the major research endpoint.
Smith et al. [19]	2018	RCT	Cochrane risk of bias assessment tool	FCU4Health is tested in this hybrid effectiveness-implementation RCT.	This research might decrease childhood obesity, overweight, and health inequities by proving FCU4Health works.
Ojeda et al. [20]	2018	RCT	Cochrane risk of bias assessment tool	An RCT was conducted with 107 people, splitting them into two groups: one got standard treatment, which consisted of a 30-minute individual session with the dietician, while the other followed a moderately hypocaloric Mediterranean diet and received nutritional counseling.	Both groups saw substantial glucose, BMI-SDS, and total cholesterol reductions.
Rohde et al. [21]	2017	RCT	Cochrane risk of bias assessment tool	Out of 635 Danish preschoolers with birth weights ≥4000 g, 285 completed the intervention and provided full nutritional consumption data.	After 15 months of intervention, children consumed less energy.
Händel et al. [22]	2017	RCT	Cochrane risk of bias assessment tool	35 normal-weight study subjects were selected between two- and six-year-old preschoolers from Greater Copenhagen.	According to the findings of the linear regression analysis, the intervention group spent more time per week participating in sports and outdoor activities than the control group did in the post-intervention evaluation.
Benestad et al. [23]	2017	RCT	Cochrane risk of bias assessment tool	Lifestyle school with 4 days of family education or a 2-week summer camp with 4 repeat weekends.	Summer camp kids had a smaller adjusted projected mean BMI gain, but their BMI SDS decreases were similar.
Norman et al. [24]	2016	RCT	Cochrane risk of bias assessment tool	Obese 11-13-year-olds from San Diego primary care clinics were studied.	No biometric or adiposity differences were identified across groups.
Foley et al. [25]	2016	RCT	Cochrane risk of bias assessment tool	The family chosen for the study used SWITCH intervention RCT to reduce the time spent screening overweight patients between nine and 12 years old.	Poor uptake and intervention effectiveness may have contributed to the SWITCH trial's null outcomes on health behavior and body composition.
Taveras et al. [26]	2015	RCT	Cochrane risk of bias assessment tool	549 study subjects between six and 12 years old with a 95% percentile BMI or above from 14 Massachusetts primary care clinics from October to June	The three arms exhibited varying impacts on BMI over time. BMI grew less in CDS children than in standard-care children in the first year.
Kobel et al. [27]	2014	RCT	Cochrane risk of bias assessment tool	The cluster RCT included 43 children (7.1 ± 0.6 years) who were examined, and 1736 of them did follow-up.	The analysis did not reveal any substantial distinctions between SDC and PA.

Meta-Analysis of Outcomes

**Figure 2:**
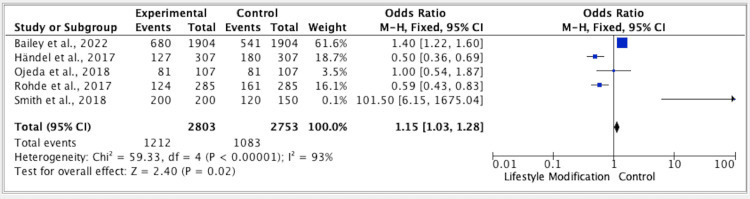
Forest plot for studies about the efficacy of lifestyle modification vs. control group M-H: Mantel-Haenszel; CI: confidence interval. References: [17,19-22].

The results of five studies showed a p-value of 0.01, a CI of 1.03-1.28, an OR of 1.15 (FE, 95%), and an I^2^ of 93% for the efficacy of lifestyle modification vs. the control group (Figure 2).

**Figure 3:**
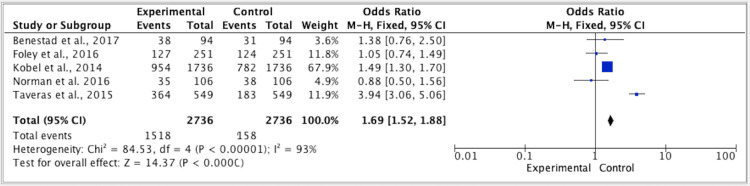
A forest plot for studies about the efficacy of lifestyle modification vs. control group M-H: Mantel-Haenszel; CI: confidence interval. References: [23-27].

The results of five studies showed a p-value of 0.01, a CI of 1.52-1.88, an OR of 1.69 (FE, 95%), and an I^2^ of 93% for the efficacy of lifestyle modification vs. the control group (Figure 3).

**Figure 4:**
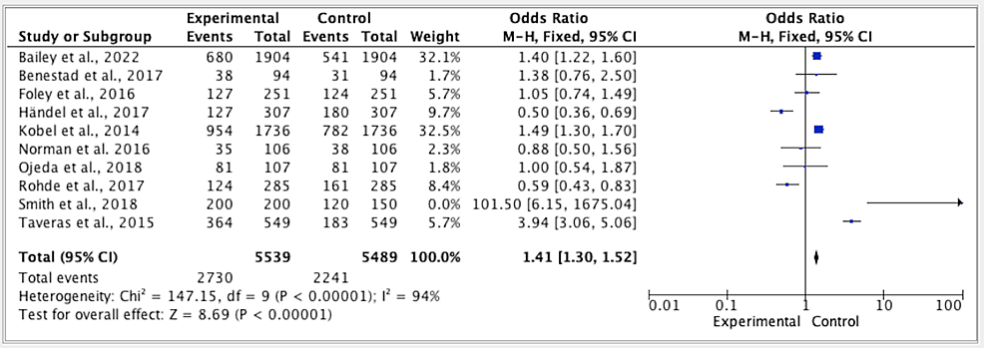
Forest plot for studies about the overall efficacy of lifestyle modification vs. control group M-H: Mantel-Haenszel; CI: confidence interval. References: [17,19,20-27].

The results of five studies showed a p-value of 0.01, a CI of 1.30-1.52, an OR of 1.41 (FE, 95%), and an I^2^ of 94% for the efficacy of lifestyle modification vs. the control group (Figure 4).

**Figure 5:**
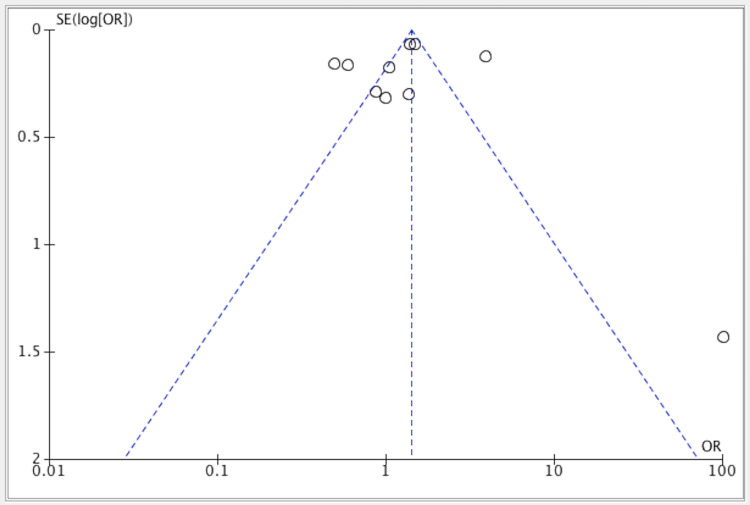
Funnel plot for all included studies about the efficacy of lifestyle modification vs. control group References: [17,19,20-27].

Publication bias was seen in four studies, while it was not seen in six of them (Figure 5). 

Discussion

In a large study by Bailey et al., parents showed the efficacy of the PRO tool by using it through the patient portal or on their own device, tablet, or kiosk during their clinic visit [17]. These parents not only received timely feedback but also had the opportunity to select the most relevant topics for discussion with their healthcare provider [17]. Bailey et al. seamlessly integrated the study’s outcomes into the child’s electronic health record, enabling healthcare professionals to deliver personalized preventive counseling. PRO-enhanced WCVs made it easier for families to connect with community health experts. These experts then used evidence-based telehealth interventions to help fight obesity and manage food resources after the WCV [17]. The primary objective of this research was to assess the impact of World Health Organization growth standards on the child’s BMI z-score one year after the baseline WCV [17].

Smith et al. conducted an important RCT to address another pressing issue in the management of childhood obesity: the lack of evidence-based programs in primary health care [19]. The U.S. Preventive Services Task Force engaged in a study focusing on family-centered weight management interventions for children aged 6-12 who were either overweight or obese. The main goal of this study was to look into all the problems that arise when you try to use a complete, evidence-based program in the real world [19]. The success of Family Check-Up 4 Health (FCU4Health) could potentially have a significant impact on the pediatric obesity epidemic and could address healthcare disparities related to chronic conditions [19].

Ojeda et al. conducted a separate study where they took two groups: a specialized group that consisted of a doctor follow-up in metabolic analysis, a moderate hypocaloric Mediterranean diet, and nutritional education, while the usual care group, which consisted only of a follow-up visit of 30 minutes without a moderately hypocaloric Mediterranean diet and nutritional education, showcased the effectiveness of a comprehensive lifestyle intervention for children and adolescents dealing with abdominal obesity [20]. They found this intervention greatly decreased BMI standard deviation score (BMI-SDS) and made people more likely to follow dietary advice, as shown by diet quality indices. Furthermore, the study revealed a positive correlation between higher diet quality scores and improved micronutrient adequacy [20].

In another study, Rohde et al. focused on addressing obesity in children aged 2-6 years, a group particularly susceptible to this condition [21]. They uncovered a significant reduction in the overall energy intake among children who underwent the intervention over a 15-month period [21]. Importantly, there were no significant differences in macronutrient composition or food consumption before and after the intervention. However, the intervention had an impact on energy intake, primarily due to reduced carbohydrate and added sugar consumption [21]. Reducing total energy intake carries the potential benefit of mitigating the risk of excessive weight gain and potentially preventing the development of obesity. Additionally, lower consumption of added sugar signifies improved diet quality [21].

Händel et al. focused on the Healthy Start primary intervention, which aimed to increase the amount of time parents spend engaging in sports and outdoor activities [18]. This increase served as a proxy for measuring the level of moderate to vigorous physical activity. Conversely, Benestad et al. did not find any specific effects for different types of physical activity, such as playing sports, spending time outside, using screens, or frequency of commuting [23]. Unlike outpatient programs, a two-year family camp-based obesity treatment program showed limited long-term effects on BMI and certain cardiovascular risk factors in children with severe obesity. Moreover, a significant number of children continued to experience obesity following their treatment, raising questions about the cost-effectiveness of summer camps [23].

Norman et al. investigated the efficacy of a stepped-down intervention given by clinician and health educator counseling (in-person and by phone) and mailed material. The 'steps' of the intervention lasted for four months, with the first month being the most intense and the last month being the least demanding if the treatment objectives were achieved. Patients in the EUC group were offered a one-time appointment with a doctor, a health counseling session, and regular mail-outs of educational materials. However, there was a statistically significant treatment impact on BMI (p 0.001) only for males [24]. The fact that boys’ BMI decreased after the intervention is encouraging and it suggests that personalized programs for dealing with obesity in primary care settings should be used based on behavioral theory [24].

## Conclusions

In most of the studies included in our analysis, researchers carefully selected a diverse and extensive population. To ensure the reliability and completeness of the data, researchers engaged parents in various settings, allowing for a comprehensive exploration of preventive strategies against childhood obesity. One of the standout interventions in our findings is FCU4Health. This approach has shown great promise in addressing pediatric obesity, particularly in cases involving chronic conditions. It reduces child BMI-SDS and promotes healthier nutritional habits. Significant benefits were there when there was a reduction in the consumption of carbohydrate-rich and sugary foods. This not only helped prevent excessive weight gain but also enhanced the overall quality of the diet, contributing to improved health. Encouraging children to engage in outdoor physical activity emerged as a common and highly effective recommendation. This approach yielded numerous health benefits, particularly in terms of reducing BMI and mitigating cardiovascular risk factors.
